# Molecular and clinical characteristics of IDH mutations in Chinese NSCLC patients and potential treatment strategies

**DOI:** 10.1002/cam4.4764

**Published:** 2022-05-08

**Authors:** Shuchen Chen, Honglin Zhu, Meizi Jin, Hongling Yuan, Zhenzhen Liu, Jielin Li, Xiang Zhang, Lihui Meng, Ting Li, Yuzhu Diao, Hong Gao, Chengyu Hong, Xinjiang Zhu, Jian Zheng, Fei Li, Yanling Niu, Tonghui Ma, Xiaoling Li

**Affiliations:** ^1^ Cancer Hospital of China Medical University Shenyang China; ^2^ Liaoning Cancer Hospital & Institute Shenyang China; ^3^ Hangzhou Jichenjunchuang Medical Laboratory, Co., Ltd. Hangzhou China

**Keywords:** IDH inhibitor, *IDH1*, *IDH2*, next‐generation sequencing, non‐small‐cell lung cancer

## Abstract

**Background:**

Isocitrate dehydrogenase (IDH) is an appealing target for anticancer therapy, and IDH (IDH1/2) inhibitors have been approved for targeted therapy of acute myeloid leukemia (AML) and Cholangiocarcinoma. The therapeutic potential of IDH inhibitors for non‐small‐cell lung cancer (NSCLC) patients is under active clinical investigation. Thus, it would be necessary and meaningful to study the molecular and clinical characteristics of IDH mutation in NSCLC patients, especially in the Chinese population.

**Methods:**

A total of 17,978 Chinese patients with NSCLC who underwent next ‐generation sequencing (NGS) testing were retrospectively reviewed.

**Results:**

We identified 161 unique IDH mutations in 361 of 17,978 patients (2.01%). Common active‐site mutations, including IDH1^R100^, IDH1^R132^, IDH2^R140^, and IDH2^R172^, were detected in 154 patients (0.86%) and were associated with male sex (*p* = 0.004) and older age (*p* = 0.02). The IDH mutation spectra observed in NSCLC were quite different from those in glioma or AML. Patients with IDH active‐site mutations exhibited significantly higher coalterations in KRAS (p. G12/13/61, 22.1% vs. 8.2%, *p* < 0.001) or BRAF (p. V600E, 6.5% vs. 1.0%, *p* < 0.001), but significantly lower coalterations in activating EGFR (e18–e20, 22.7 vs. 37.9%, *p* < 0.001) than IDH wild‐type patients. Furthermore, we found that active‐site IDH mutations were correlated with a short PFS (2–5.6 months) and short OS (2–9.5 months), which may arise as a resistance mechanism against common targeted drugs. In vitro, we experimentally observed that the combination of an IDH inhibitor and EGFR TKI could better inhibit lung cancer cell proliferation than an EGFR TKI alone.

**Conclusions:**

Taken together, this study reveals the molecular and clinical characteristics of IDH mutations in Chinese NSCLC patients and provides a theoretical basis for IDH‐directed treatment. The potential of IDH mutations as response markers for targeted therapy warrants further investigation.

## Graphical abstract

we performed a systematic analysis of clinical and molecular characteristics of IDH mutations in Chinese patients and obtained some clinical practice points.

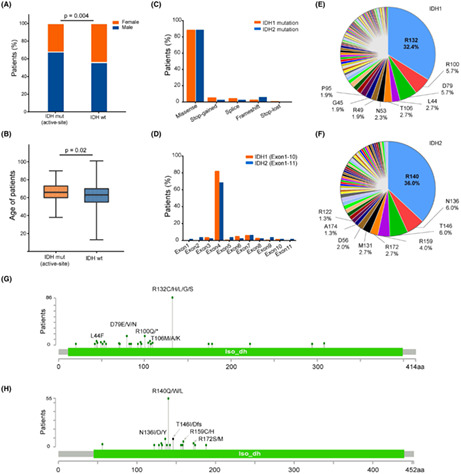

## 1 INTRODUCTION

Isocitrate dehydrogenase 1 and 2, termed IDH1 and IDH2, respectively, are key metabolic enzymes that catalyze isocitrate to α‐ketoglutarate (αKG) in the tricarboxylic acid cycle.^1^ IDH mutations confer a neomorphic enzyme activity for the reduction of αKG to D‐2‐hydroxyglutarate (D‐2HG).^2^, ^3^ D‐2HG is thought to be an oncometabolite that drives the formation of cancers in a variety of tissues, as it alters the epigenetic state of progenitor cells and impairs cell differentiation by inhibiting enzymes that regulate histone and DNA demethylation.^4^, ^5^

Mutations in IDH1 were first reported in a genome‐wide analysis of patients with glioblastomas.^6^ Subsequent studies revealed that mutations in IDH1 or IDH2 are prevalent in a variety of cancer types, including low‐grade glioma and secondary glioblastoma (~80%),^7^, ^8^ acute myeloid leukemia (AML, ~20%),^9^, ^10^ and intrahepatic cholangiocarcinoma (~10%).^11^, ^12^ Recent studies have found that IDH mutations occur in NSCLC at a relatively low rate (0.4%–1.1%)^13^, ^14^ but represent an important and promising therapeutic target.

In recent years, IDH mutations have become an attractive target for cancer therapy. A growing number of preclinical studies and clinical trials have demonstrated that IDH inhibitors can normalize intracellular D‐2HG levels.^15^, ^16^, ^17^ Consequently, IDH1 (ivosidenib) and IDH2 (enasidenib) inhibitors have been approved by the US Food and Drug Administration (FDA) for targeted therapy of AML, and ivosidenib has recently been approved for locally advanced or metastatic cholangiocarcinoma.^18^, ^19^, ^20^ Currently, several clinical trials using IDH1/2 inhibitors, such as NCT04056910 (phase II study of IDH1 inhibitor ivosidenib and nivolumab in IDH1‐mutant gliomas and advanced solid tumors), NCT02746081 (phase I study of BAY1436032 in IDH1‐mutant advanced solid tumors), and NCT02481154 (phase I study of orally administered AG‐881 in IDH1/IDH2‐mutant advanced solid tumors), have been conducted to explore the efficacy of these treatments against advanced solid tumors including NSCLC.^4^ The preliminary findings from these clinical trials may transform treatment strategies for IDH‐mutated patients of multiple tumor types. In the near future, the results of ongoing and future clinical studies may help to guide the clinical decision‐making process of patients to ensure the patients receive the best combination therapy.

Next‐generation sequencing (NGS) can identify novel potential lung cancer targets, such as translocations in the MET, RET, and NTRK family genes (NTRK1, NTRK2, and NTRK3), and has been used to significantly expand the clinical application of targeted therapies.^21^, ^22^ However, little is known about the application of IDH‐mutant inhibitors in NSCLC patients. Due to the increasing number of NSCLC patients with IDH mutations detected by NGS and the results of a series of clinical trials involving patients with IDH mutations, mutant IDH has emerged as an ideal therapeutic target for lung cancer. Therefore, it is necessary to clarify the clinicopathological characteristics of these patients and investigate the clinical application of IDH‐directed therapy.

In the present study, we performed a systematic analysis of IDH mutations in a large number of Chinese patients with NSCLC and preliminarily explored the role of IDH inhibitors in lung cancer cells through in vitro experiments.

## 2 MATERIALS AND METHODS

### 2.1 Patients and sample collection

A series of 17,978 lung cancer and 6319 glioma clinical cases who underwent NGS testing in a Clinical Laboratory Improvement Amendments (CLIA)‐certified laboratory (Genetron Health Technology, Beijing, China) between January 2017 and June 2020 were retrospectively analyzed in this study. All included patients had signed informed consent before sample collection. Acute myeloid leukemia (AML) data set (OHSU, Nature 2018), with a total of 672 samples, was downloaded from *cbioportal* (http://www.cbioportal.org/).

### 2.2 DNA extraction and NGS library preparation

Total DNA from tumor tissues was extracted using the QIAamp DNA Tissue Kit (Qiagen) according to the manufacturer's instructions. Cell‐free DNA was extracted from plasma using the MagMAX™ CellFree DNA Isolation Kit (ThermoFisher Scientific). All isolated DNAs were quantified with the Qubit 2.0 Fluorometer according to the recommended protocol. Qualified DNA samples were subjected to NGS library preparation, and then prepared DNA libraries were captured with custom tumor panels. Captured samples were subjected to 150 bp paired‐end sequencing using Illumina Hiseq X‐Ten.

### 2.3 NGS data analysis

Raw reads were preprocessed using Trimmomatic (v0.36) to remove sequence adapters and low‐quality regions, and then filtered reads were aligned to the reference genome (hg19) with BWA (v0.7.10). Mutation analysis for somatic single nucleotide variants (SNV), short insertion and deletions (InDels), and copy number alterations (sCNAs) were performed using muTect,^23^ Strelka, ^24^, ^25^ and ADTEx,^26^ respectively. All mutations were annotated with Oncotator and Variant Effect Predictor (VEP). For single nucleotide variants, a cutoff value of >1% was chosen for tissue samples and >0.1% for ctDNA samples.

### 2.4 Cell lines and reagents

HCC827 cells were a gift from the College of Life and Environmental Sciences, Hangzhou Normal University. Erlotinib Hydrochloride and AGI‐5198 were purchased from APExBIO and used as the final concentration of 2 μM (Erlotinib) and 10 μM (AGI‐5198). Anti‐IDH1 R132H was purchased from Merck (SAB4200548) and used with the dilution of 1:1000, anti‐β‐actin was purchased from BOSTER (BM0627) and used with the dilution of 1:1000. The secondary antibody was HRP‐conjugated affiniPure goat anti‐mouse IgG (H + L) (1:5000, BA1051, BOSTER).

### 2.5 Lentiviral expression constructs and transduction

IDH1‐WT was PCR cloned from a human IDH1 ORF clone construct (Youbio) with two restriction enzymes BamHI and XhoI and then were cloned to the virus vector PMT406 (APPLEGOO BIOTECHNOLOGY). IDH1‐R132H was directly synthesized and constructed into the virus vector PMT406. These plasmids were cotransfected with pCMV‐dR8.91 (Addgene) and pCMV‐VSV‐G (Addgene) into 293 T cells to produce lentivirus. Viral supernatants were collected 48 h after transfection and added to HCC827 cells in the presence of 8 μg/ml polybrene (Millipore). Infected cells were selected with puromycin treatment (2 μg/ml) and then were used for the following experiments.

### 2.6 Immunoblotting

Cells were harvested and lysed in RIPA buffer (Beyotime), and protein concentration was calculated with a BCA protein concentration detection kit (Beyotime). Thirty micrograms of total protein of each sample were separated with SDS‐PAGE, transferred to PVDF membrane (Millipore), and stained with indicated antibodies. Immunoblot was detected and exposed on a film.

### 2.7 Cellular proliferation

Cells were seeded in 96‐well plates at a dose of 5 × 10^3^ cells/well. The day following seeding, the indicated dose of drug or inhibitor was added. After 48 h, the CCK‐8 solution (MedChem Express) of 10 μl was added to each well. After 4 h of incubation, absorbance at 490 nm was measured using a microplate reader (Molecular Devices).

### 2.8 Statistical analysis

Statistical analyses were performed using R version 3.6.2 (http://www. r‐project.org) and GraphPad Prism version 7.01 (https://www.graphpad.com/). Comparisons between two groups were performed by paired Student *t* test or unpaired Student *t* test, as appropriate. For comparison of proportion values, Fisher exact test was used.

## 3 RESULTS

### 3.1 Somatic IDH1/2 mutations in NSCLC patients

**FIGURE 1:**
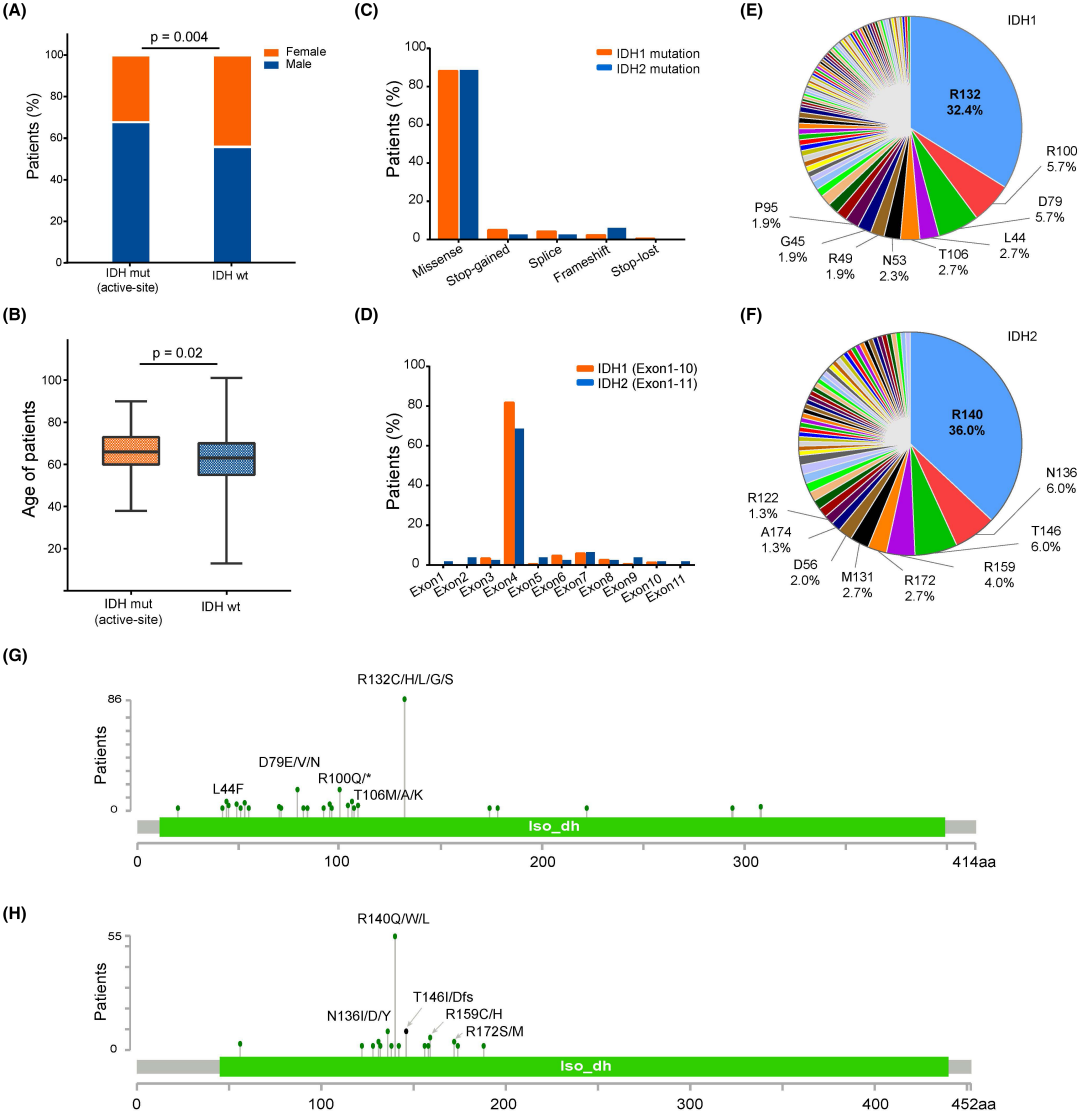
Somatic mutation of IDH in NSCLC. (A) Sex distribution of patients with and without IDH active‐site mutations. (B) Age distribution of patients with and without IDH active‐site mutations. (C) Mutation category of 412 IDH1/2 mutation sites in NSCLC. (D) Exon location of IDH1/2 mutations in the current cohort. (E, F) Distribution of the protein changes in IDH1/IDH2 mutations in NSCLC. (G‐H) A lollipop mutation diagram for IDH1/IDH2 protein mutations. Mutations shown in the diagram are the ones detected in more than one patient (*n* ≥ 2) in the current cohort

We identified 412 IDH mutations in 361 of the 17,978 patients. Of these, active‐site mutations in IDH1 and IDH2 (IDH1^R100^, IDH1^R132^, IDH2^R140^, and IDH2^R172^) were detected in 154 patients (0.86%), including 103 (66.9%) males and 51 (33.1%) females (Figure 1A). The median age of the patients with IDH active‐site mutations was 65 years old (age range 33–92 years), which was significantly older than those with wild‐type IDH (IDH‐WT) (Figure 1B). Among the 412 IDH mutations, missense variations were the most frequent mutation categories (88.2% and 88.7% for IDH1 and IDH2, respectively). Other nonsynonymous mutations occurred at a lower rate, including stop‐gained (IDH1:4.96%, IDH2:2.61%), splice (IDH1:4.19%, IDH2:2.67%), frameshift (IDH1:2.29%, IDH2:6%), and stop‐loss variations (IDH1:0.38%, IDH2:0%) (Figure 1C). In addition, IDH mutations mainly occurred in exon 4 (IDH1^exon4^: 81.7%, IDH2^exon4^: 68.6%) (Figure 1D). For IDH1 mutations, R132 (32.4%), was the most common mutation, followed by R100 (5.7%), D79 (5.7%), L44 (2.7%), and T16 (2.7%) (Figure 1E). For IDH2 mutations, the most common mutation was R140 (36%), followed by N136 (6.0%), T146 (6.0%), R159 (4%), and R172 (2.7%) (Figure 1F). *IDH*1/2 mutations found in ≥ 2 patients (n ≥ 2) are summarized in Figure 1G, H.

### 3.2 IDH active‐site mutations vary among NSCLC, glioma, and AML

IDH mutations are the most common oncogenic driver mutations in glioma and AML,^8^, ^27^ and IDH inhibitors approved by the FDA and those in clinical trials mainly target IDH active‐site mutations in these two cancers. As IDH inhibitors show different inhibitory effects on different mutation isoforms (amino acid substitution at arginine residue),^16^, ^20^, ^28^ we then sought to compare IDH active‐site mutation isoforms in NSCLC with those in glioma and AML. Four active‐site mutation codons were mapped to key residues within the active site, arginine, which is critical for isocitrate binding. Notably, the IDH1^R100^ codon mutation, which has been previously reported,^29^, ^30^, ^31^ exhibited a relatively higher mutation frequency in our study. Missense substitutions in four IDH active‐site mutation codons, IDH1^R132^ (*n* = 88), IDH1^R100^ (*n* = 10), IDH2^R140^ (*n* = 52), and IDH2^R172^ (*n* = 4) ranged widely from polar (C, H, L, Q, T, S, and K) to nonpolar (W and M).

**FIGURE 2:**
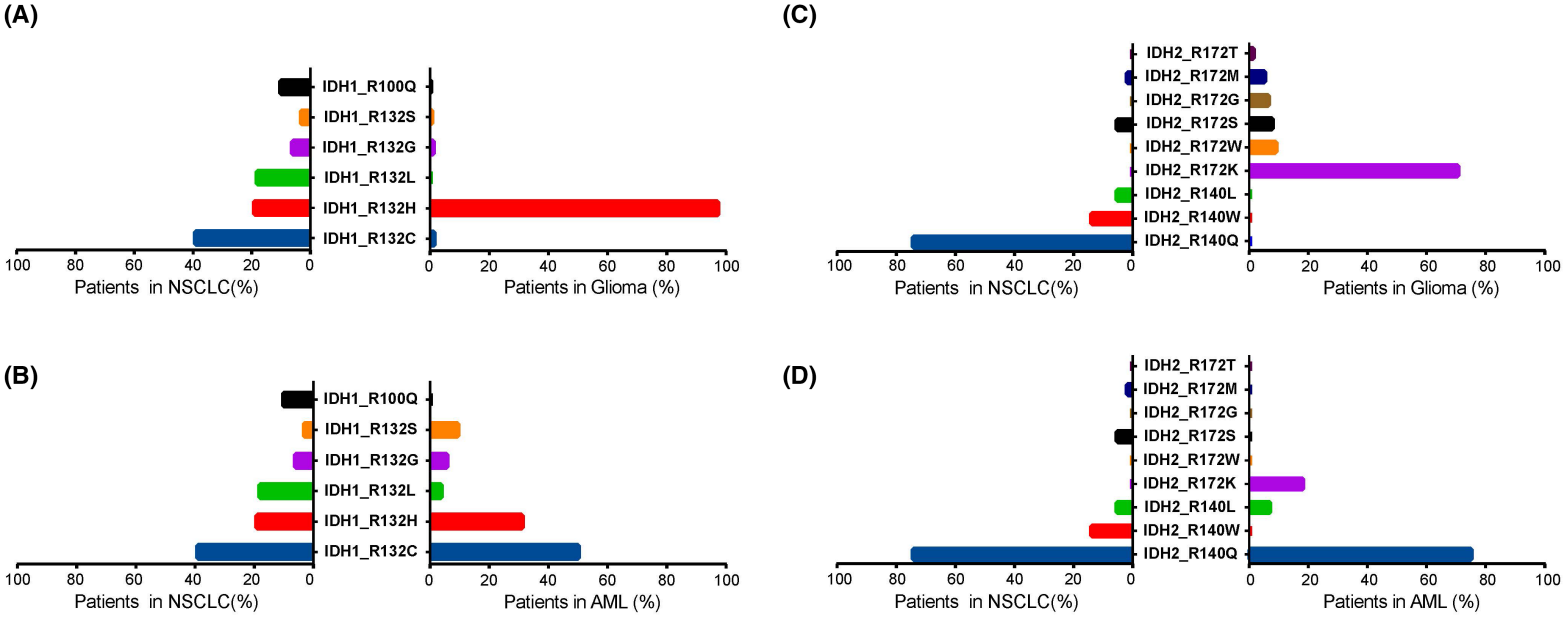
Comparison of IDH active‐site mutations among NSCLC, glioma, and AML. (A, B) Comparison of IDH1 hotspot mutations (IDH1R132 and IDH1R100) in NSCLC, with glioma (A) or AML (B). (C, D) Comparison of IDH2 hotspot mutations (IDH2R140 and IDH2R172) in NSCLC, with glioma (C) and AML (D)

Out of the 98 IDH1 active‐site mutated specimens, 41 (41.8%) carried IDH1^R132C^ mutations, followed by IDH1^R132H^ (20.4%), IDH1^R132L^ (18.4%), IDH1^R100Q^ (10.2%), IDH1^R132G^ (6.1%), and IDH1^R132S^ (3.1%). Fifty‐six patients harbored IDH2 active‐site mutations, including IDH2^R140Q^ (75.0%), IDH2^R140W^ (12.5%), IDH2^R140L^ (5.4%), IDH2^R172S^ (5.4%), and IDH2^R172M^ (1.8%). A comparison between IDH1/2 missense substitutions across four active‐site mutations among NSCLC (from Genetron, *n* = 17,978), glioma (from Genetron, *n* = 6319), and AML (from OHSU, *n* = 672) data sets^32^ revealed that IDH1 mutations in glioma were predominantly R132H (97.1%), however, they were more dispersed in NSCLC and AML (Figure. 2A, B). In addition, IDH2 active‐site mutations mainly occurred at the R172 codon site in glioma, whereas the R140 codon was the most common mutation site in NSCLC and AML (Figure. 2C, D). The rate of IDH1^R132L^ in NSCLC was significantly higher than those in glioma (*p* < 0.001) and AML (*p* = 0.008). Furthermore, *IDH1* ^R100Q^ and *IDH2* ^R140W^ mutations were found to exclusively occur in NSCLC.

### 3.3 Genomic coalterations associated with IDH

**FIGURE 3:**
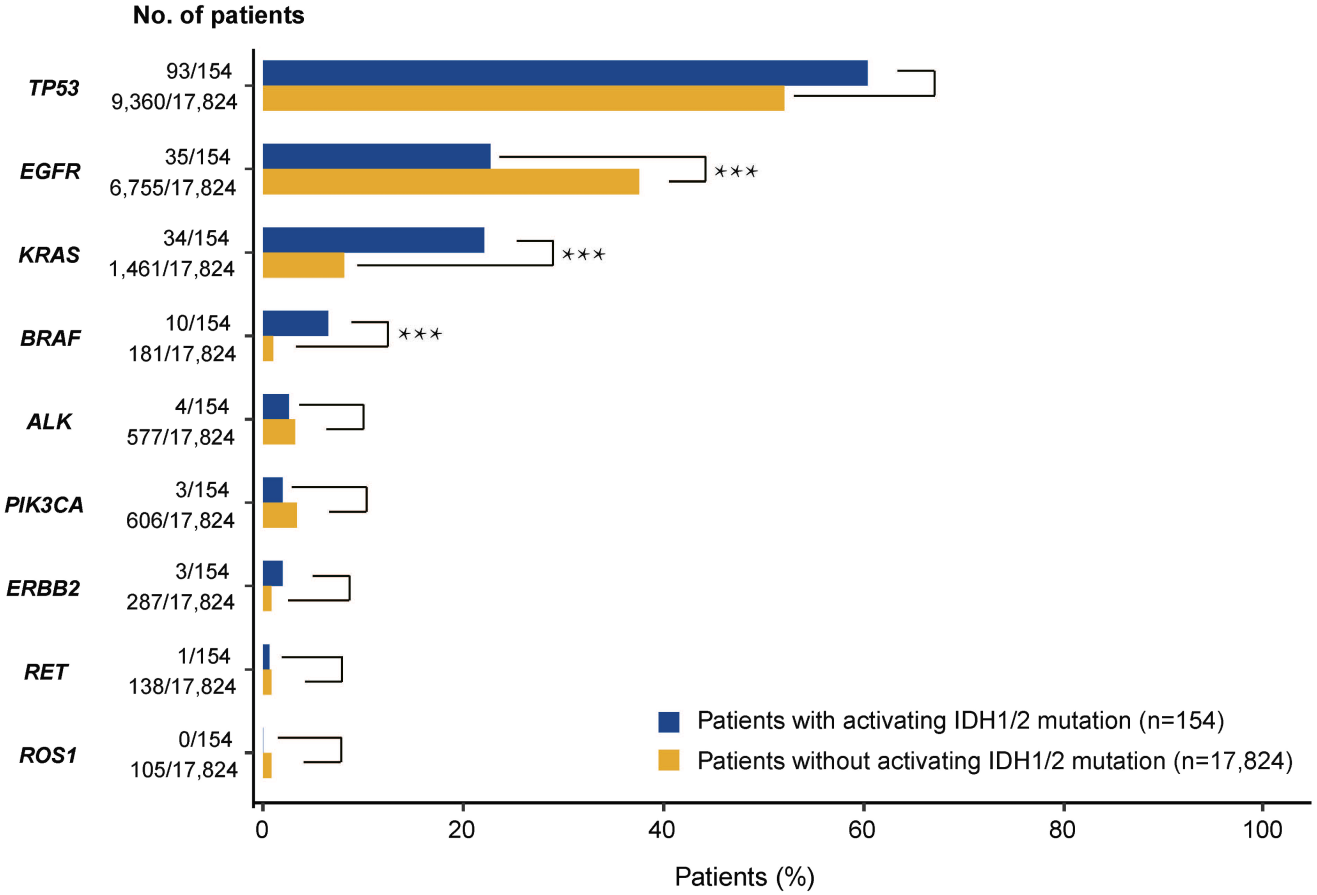
Comparison of genomic alterations in patients with (*n* = 154) and without (*n* = 17,824) IDH active‐site mutations. Eight important coalterations associated with IDH mutations are shown. Among these genes, the frequency of either KRAS or BRAF mutations was higher in IDH‐MUT tumors than in IDH‐WT tumors. The frequency of EGFR mutations, however, was lower in IDH‐MUT tumors than in IDH‐WT tumors (*p* < 0.001)

Among the 154 patients with IDH active‐site mutations (54 in tissues, 100 in ctDNAs), we identified a total of 2898 mutations. The top covariation genes were *TP53* (60.4%), *EGFR* (31.8%), *ALK* (31.8%), *KRAS* (25.3%), and *BRAF* (25.3%) (Figure S1). To understand the mutation profiles and distribution of *IDH* mutations in NSCLC, we compared the eight most common activating driver mutations in NSCLC with (*n* = 154) and without (*n* = 17,824) active‐site IDH, including *TP53* (60.4% vs. 54.8%), *EGFR* (e18–e21, 22.7% vs. 37.9%), *KRAS* (p. G12/13/61, 22.1% vs. 8.2%), *BRAF* (p. V600E, 6.5% vs. 1.0%), *ALK* (2.6% vs. 3.2%), *PIK3CA* (p. E542/E545/H1047, 1.9% vs. 3.4%), *ERBB2* (1.9% vs. 1.6%), *RET* (0.65% vs. 0.7%), and *ROS1* (0% vs. 0.6%) (Figure 3). In addition, the results from Fisher's exact test revealed that tumors carrying *KRAS* (p. G12/13/61, *p* < 0.001) and *BRAF* (p. V600E, *p* < 0.001) were significantly enriched in *IDH* active site‐mutant tumors, whereas those with *EGFR* mutations were significantly less (e18–e21, *p* < 0.001) enriched in *IDH*‐mutant tumors than in *IDH*‐WT tumors (Figure 3). As *KRAS/BRAF* is a downstream pathway of EGFR, these results suggest that IDH mutations play different roles in *EGFR*‐driven and *KRAS/BRAF*‐mediated NSCLC.

### 3.4 IDH mutations were mainly subclonal mutations in NSCLC

**FIGURE 4:**
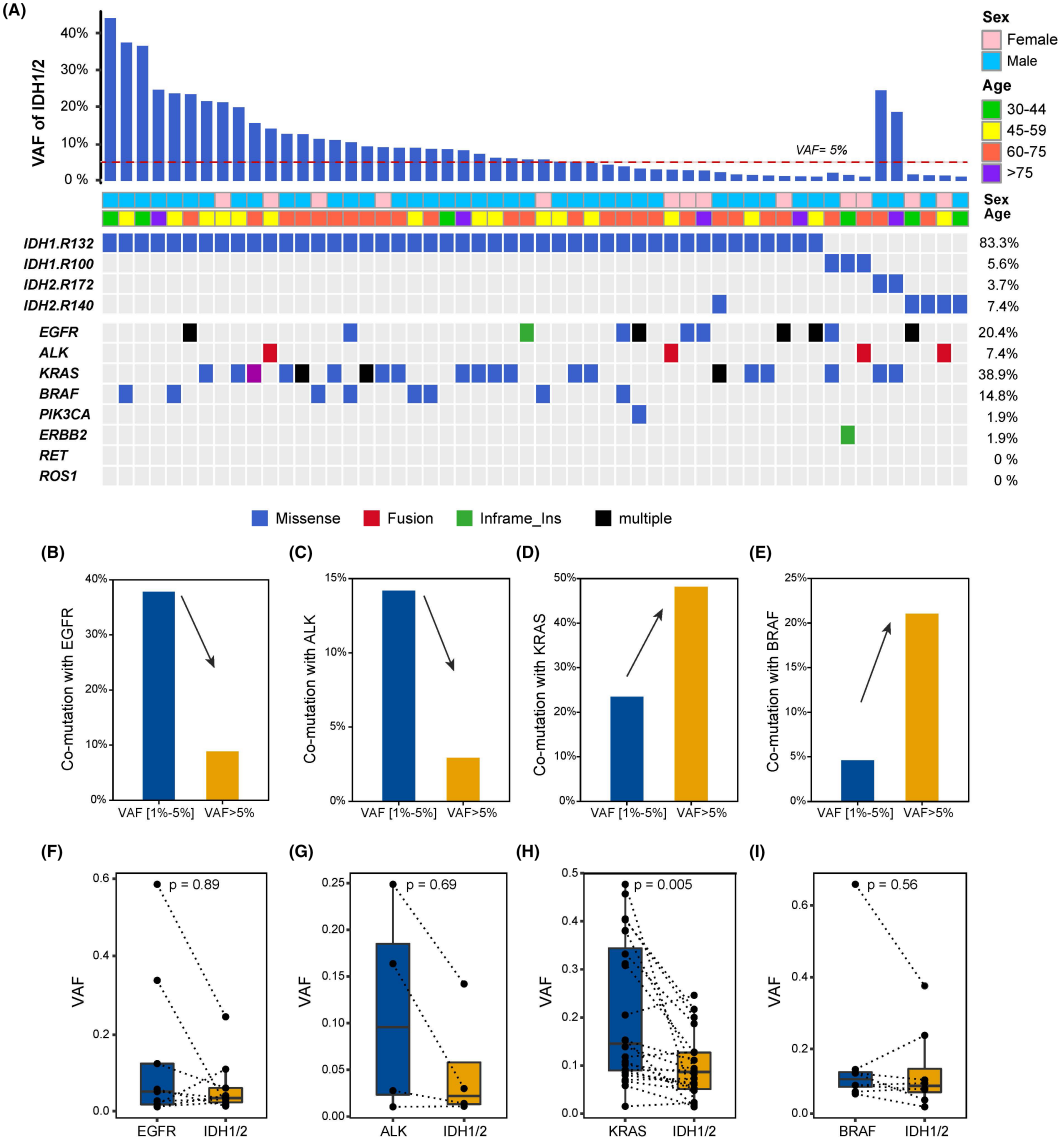
Subclonal analysis of IDH1/2 mutations. (A) Heatmap of IDH mutations and coalterations with important trunk driver mutations in NSCLC (EGFR, ALK, KRAS, BRAF, PIK3CA, ERBB2, RET, and RO3). (B–E) Coalteration with known driver genes (EGFR, ALK, KRAS, and BRAF) at different VAF levels of IDH1/2 mutations. (F–I) Comparison of VAF between IDH1/2 active‐site mutations and other known driver genes

Intratumor heterogeneity is an important factor in the efficacy of targeted therapy. To identify whether IDH mutations are primary or subclonal, we screened 54 samples harboring IDH active‐site mutations detected in tissues and compared the levels of variant allele frequencies (VAFs) between IDH mutants and other important trunk driver genes in NSCLC (Figure 4A). We found coalterations in eight driver genes in 72.2% of NSCLC patients, namely, *EGFR* (e18–e21, 20.4% [11 of 54]), *ALK* (fusion, 7.4% [4 of 54], *KRAS* (p. G12/13/61, 37% [20 of 54]), *BRAF* (p. V600E, 13% [7 of 54]), *ERBB2* (1.9% [1 of 54]), *PIK3CA* (p. E542/E545/H1047, 1.9% [1 of 54]), *RET* (0% [0 of 54]), and *ROS1* (0% [0 of 54]). In addition, we found that the VAF of IDH mutations was usually low when coexisting with *EGFR* and *ALK* alterations, and conversely, it was usually high when comutated with *KRAS* and *BRAF* mutations. When the VAF of IDH mutation was at a high level (VAF ≥ 5%, *n* = 32), we observed a decrease in coalteration of IDH with *EGFR* mutation and *ALK* fusion (*EGFR*: 34.8–9.4%, *ALK*: 13–3.2%). However, its coalteration with *KRAS* p. G12/13/61 and *BRAF* p. V600E was increased (*KRAS*: 30.4–45.2%, *BRAF*: 4.3–19.3%) (Figure 4B–E). Among the coalteration samples, the VAF of *KRAS* was significantly higher than that of IDH1/2 (*p* = 0.005) (Figure 4H).

In NSCLC, most *EGFR‐*, *KRAS‐*, and *BRAF‐*activating mutations are trunk drivers.^33^ In our study, we found that 9.5% (4/42) of VAF^IDH^ was higher than the maximum VAF of coalteration trunk driver genes (VAF^IDH^–VAF^max [EGFR/ALK/KRAS/BRAF]^ > 0.01), which was lower than the expected VAF for IDH active‐site mutations, indicating that they were likely subclonal evolution of branching drivers of lung adenocarcinoma. When not coexisting with the trunk driver genes (*n* = 12), the VAF of IDH active‐site mutations was relatively high, which suggested that they might be driver clones **(**Figure 4B–I **)**.

### 3.5 Subclonal IDH mutations show resistance to targeted therapy

**FIGURE 5:**
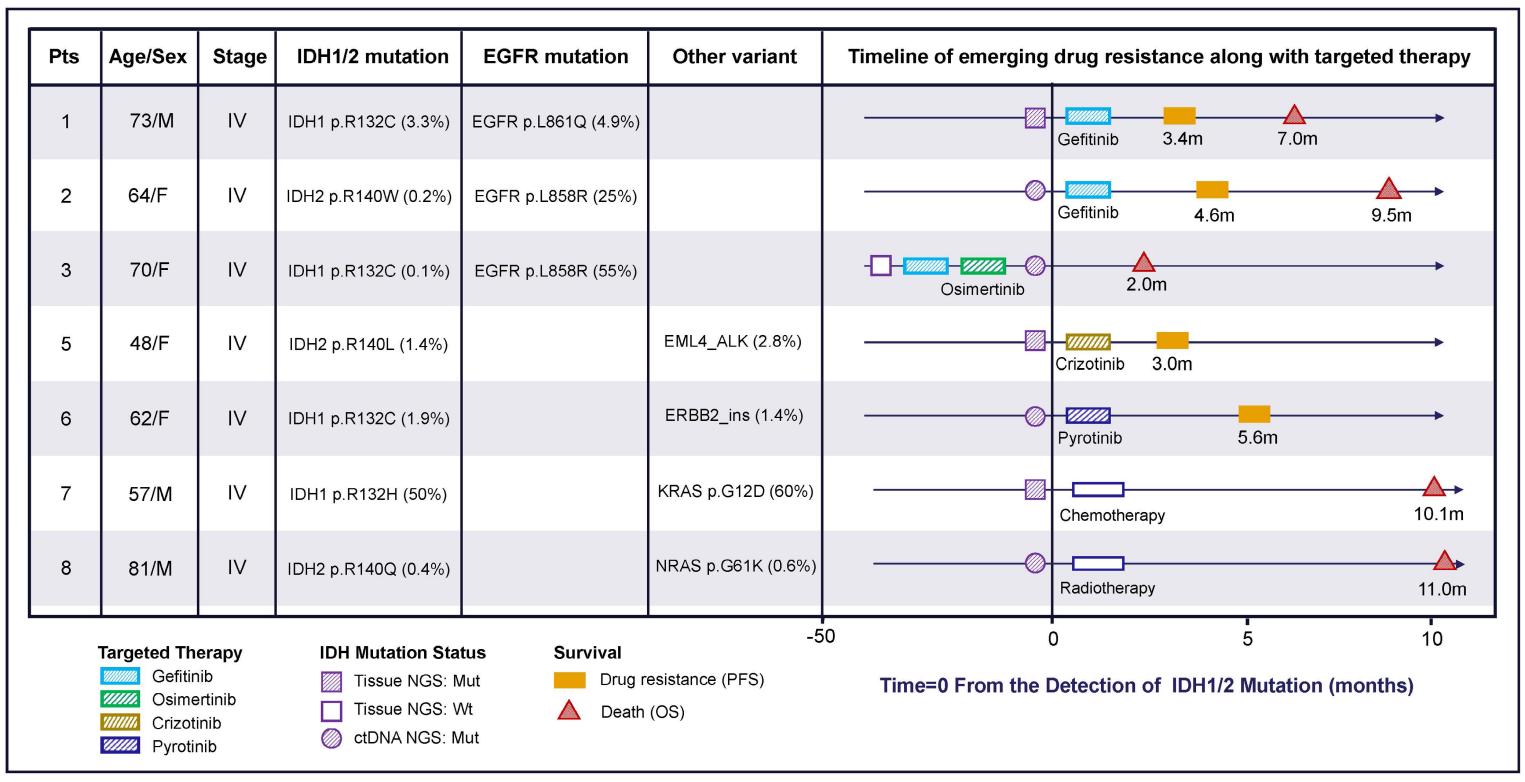
Overview of disease progression in 7 patients (stage IV) with IDH1/2 mutations. Four patients initially tested positive for IDH1/2 mutations in tissue, and 3 patients tested positive for ctDNA. IDH1/2 active‐site mutations have been associated with short PFS (2–4.6 months) and short OS (2–9.5 months) in targeted therapy

We obtained detailed treatment and survival data of 8 patients with active‐site IDH1/2 mutations and found that seven of them (87.5%) showed high‐grade features (stage IV), whereas the other patients exhibited resectable stage III lung adenocarcinoma. Among these seven stage IV patients, three with activating *EGFR* mutations were treated with EGFR tyrosine kinase inhibitors (TKIs), one carried concurrent EML4‐ALK fusion and received crizotinib treatment, one carried concurrent *ERBB2* and received pyrotinib treatment, and the other two patients without common targetable mutations received radiation or chemotherapy. As a result, the five patients who received targeted therapies exhibited short PFS (3–5.6 months) and/or OS (2–9.5 months), indicating *EGFR*‐targeted therapy is not efficient enough to control disease progression. Moreover, those two patients who received chemotherapy and radiotherapy also showed a short OS (10.1–11 months) (Figure 5 and Figure S2). In addition, we further observed the development of an acquired IDH mutation in patient 3, who was first treated with gefitinib after detection of the *EGFR* mutation at codon 858 (*EGFR* ^L858R^/*IDH*‐). After gefitinib resistance occurred, the patient was further received osimertinib. However, the *IDH1*‐R132C mutation appeared after osimertinib resistance occurred, and the patient died 2 months later, which suggested a required resistance under treatment selection. Thus, *IDH* active‐site mutations were associated with short PFS and OS and might arise as a mechanism of resistance to targeted therapies.

### 3.6 The combination of an IDH inhibitor and EGFR TKIs inhibits lung cancer cell proliferation

**FIGURE 6:**
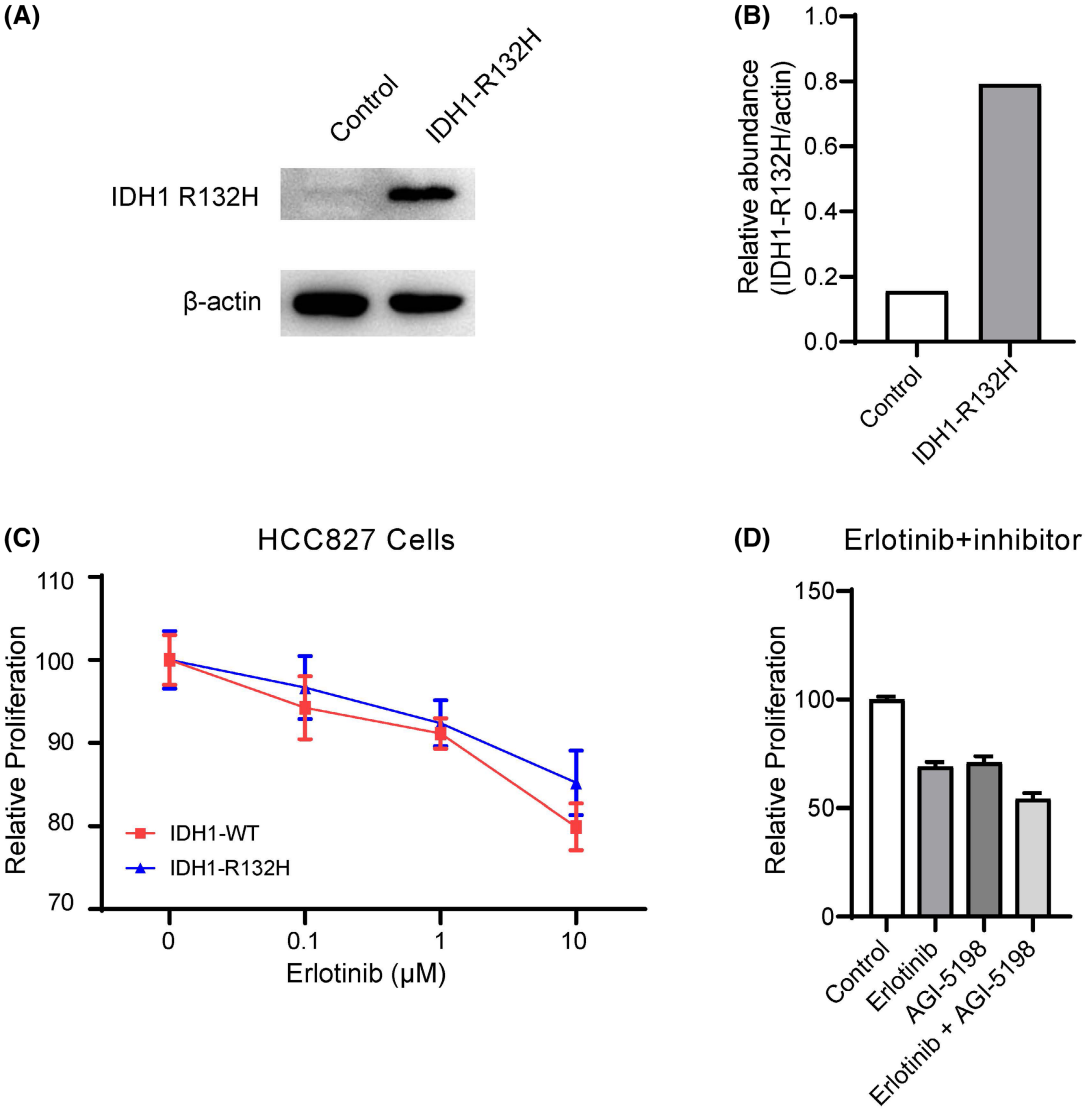
The combination of IDH inhibitor and EGFR TKIs could better inhibit lung cancer cell proliferation. (A) Immunoblot analysis of IDH1 R132H and β‐actin (loading control) in HCC827 cells infected with virus‐containing empty vector (control) or IDH1‐R132H vector. (B) Quantified IDH1‐R132H protein abundance in (A). (C) Cell proliferation analysis of HCC827 cells overexpressing wild‐type (IDH1‐WT) or mutant (IDH1‐R132H) IDH1 treated with the indicated dose of erlotinib. Data were shown as the mean ± S.E.M. (D) IDH1‐R132H overexpressing HCC827 cells were treated with Erlotinib (2 μM), AGI‐5198 (10 μM), or a combination of erlotinib and AGI‐5198. Data were shown as the mean + S.E.M

To determine whether *IDH*‐mutant NSCLC patients could benefit from the combination of IDH inhibitors and EGFR TKIs, we overexpressed wild‐type or mutant (*IDH1*‐R132H) IDH1 in HCC827 cells carrying an *EGFR* mutation in vitro. Immunoblot analysis confirmed increased *IDH1*‐R132H in HCC827 cells (Figure 6A, B and Figure S3). Then, different doses of an EGFR TKI (erlotinib) were used to treat wild‐type or mutant IDH1 overexpressing HCC827 cells. We found that the EGFR TKI efficiently inhibited HCC827 cell proliferation, as these cells contained an *EGFR* mutation. In addition, HCC827 cells overexpressing mutant IDH1 protein showed a trend of resistance to erlotinib compared with those overexpressing wild‐type IDH1 protein (Figure 6C). Next, we treated these cells with erlotinib, the IDH1 inhibitor AGI‐5198, and a combination of Erlotinib and AGI‐5198. As shown in Figure 6D, we found that both EGFR TKI and IDH inhibitor suppressed the growth of HCC827 cells and that the combination of an EGFR TKI and IDH inhibitor suppressed its growth to a lower level, indicating that the combination of an IDH inhibitor and EGFR TKI is more efficient in inhibiting tumor cell growth than the single treatment. Thus, these in vitro results revealed that EGFR‐mutant NSCLC patients who carry an IDH mutation may benefit from combined therapy of EGFR TKIs and IDH inhibitors.

## 4 DISCUSSION

In the present study, analysis of nearly 18,000 NSCLC specimens revealed a 2.01% rate of IDH mutations in Chinese patients, which was significantly higher than that in European and American populations (1.1%) reported in the TCGA and may define a subset of Chinese NSCLC patients. IDH active‐site mutations are associated with male sex, older age, coexisting KRAS (p. G12/13/61) mutation, and high‐grade features in NSCLC.

Active‐site mutations of IDH result in a gain of function producing 2‐hydroxyglutarate (2HG) from isocitrate instead of α‐KG^3^. The active IDH mutations are mainly single arginine substitutions, predominantly affecting arginine 132 (R132) or arginine 100 (R100) residue in IDH1 and arginine 172 (R172) or arginine 140 (R140) residue in IDH2.^31^, ^34^ In low‐grade gliomas, IDH1^R132H^ mutation is the most common (~90%), while in AML, the most frequent mutation is IDH2^R140Q^. In our study, it was found that there were more IDH mutation isoforms (amino acid substitution at arginine residue) in NSCLC, including IDH1^R132C^, IDH1^R132H^, IDH1^R132L^, IDH1^R132G^, IDH1^R132S^, IDH1^R100Q^, IDH2^R140Q^, IDH2^R140W^, IDH2^R140L^, IDH2^R172S^, and IDH2^R172M^. Furthermore, IDH1^R100Q^ and IDH2^R140W^ mutations were observed to exclusively occur in NSCLC. In addition, current IDH inhibitors are designed to target different groups of mutation isoforms (amino acid substitution at arginine residue). For example, BAY‐1436032 has been shown to inhibit D‐2HG production by the R132H, R132C, R132G, R132S, and R132L variants of IDH1,^31^, ^35^ whereas DS‐1001b was designed to robustly inhibit D‐2HG production by IDH1‐R132H and IDH1‐R132C. Thus, more IDH mutation isoforms need to be considered in the design of future clinical trials.

Intratumor heterogeneity is an important factor affecting the efficacy of targeted therapy. Somatic mutations include driver and subclonal mutations. Among them, driver mutations initiate the development of cancer cells, whereas subclonal mutations are progressively produced.^36^, ^37^ In the present study, the comparison of VCF between IDH mutations and coexisting known driver mutations, such as EGFR, ALK, KRAS, and BRAF, suggested that IDH1/2 mutations were mainly branching drivers. Therefore, IDH‐targeted therapies could be considered in combination with these common targetable mutations in the future. In addition, KRAS (p. G12/13/61)‐mutated NSCLCs showed a worse prognosis, responded poorly to chemotherapy, and presented at a more advanced stage relative to tumors with wild‐type KRAS.^38^, ^39^, ^40^ Our results also revealed that IDH active‐site mutations significantly co‐occurred with KRAS (p. G12/13/61) mutation, which was consistent with previous studies.^13^, ^14^ Thus, we anticipate that the identification of patients with KRAS and IDH comutations may benefit from IDH‐targeted therapy.

The implications of IDH1/2 mutations vary greatly among different cancer types. For example, IDH1 mutations have been associated with better outcomes in low‐grade gliomas.^8^ Conversely, in AML and myelodysplastic syndromes, patients with IDH1 mutations have shown poorer overall survival than IDH‐WT patients.^32^ In our study, IDH active‐site mutations might act as a primary and acquired resistance mechanism to common targeted drugs, evidenced by their association with a shorter PFS and OS after targeted therapy, chemotherapy, and radiation *therapy*. In addition, we experimentally demonstrated that the combination of IDH inhibitors and EGFR TKIs inhibited lung cancer cell proliferation, which provided novel clues for the clinical management of IDH mutants and especially targeted therapy‐resistant patients. More data from patients are needed to better evaluate the clinical outcomes of combination therapy with IDH inhibitors in IDH‐mutant lung cancer patients. Previous studies have shown that IDH‐mutant proteins can inhibit TET2 activity, while TET2 knockdown confers resistance to EGFR inhibitors in lung cancer cells.^41^, ^42^, ^43^ We hypothesized that TKIs resistance in IDH mutation patients may be related to this phenomenon, or there may be other mechanisms resulting in. Recent work suggests that IDH1 mutation promoted lung cancer cell proliferation through methylation of Fibulin‐5.^44^ Further studies are expected to fully elucidate the mechanisms of tumorigenesis and drug resistance and help guide the development of appropriate therapeutic strategies.

Accumulating evidence suggests that tumors carrying IDH mutations may be sensitive to IDH inhibitors. Two IDH inhibitors, Idhifa and Tibsovo, have been approved by the US Food and Drug Administration (FDA) for the treatment of AML or Cholangiocarcinoma.^45^ Currently, several clinical trials evaluating treatment therapies of IDH inhibitors against NSCLC are ongoing. Specifically, various other approaches, including hypermethylated drugs, kinase inhibitors, immunotherapy, and combination therapy, are being evaluated.^2^ Although the incidence of IDH mutations is uncommon in NSCLCs, considering the high prevalence and low survival rate of lung cancer in China and worldwide, tumors‐harboring IDH mutations need further investigation for the subsequent development of novel therapeutic strategies.

This study has limitations inherent in retrospective studies. Our research data lacked detailed clinical data, such as pathological stage, smoking status, and survival, which could more accurately describe the characteristics of NSCLC patients with IDH mutation. IDH mutation was associated with the resistance to targeted therapy and poor prognosis of NSCLC patients, the results were based on case analysis. In addition, we have not identified which of the other IDH mutations are active, which is very important for clinical application and we hope this will be done in our future studies.

## 5 CONCLUSION

IDH‐mutated lung cancer accounts for 2.01% of NSCLC and is associated with male sex, older age, coexisting KRAS (p. G12/13/61) mutation, and high‐grade features. IDH mutation spectra observed in NSCLC were quite different from those in glioma or AML, and IDH mutations in NSCLC were mostly branching drivers leading to subclone evolution. Furthermore, IDH active‐site mutations were correlated with a short PFS and OS, which may arise as a resistance mechanism against commonly targeted drugs. In vitro, we experimentally demonstrated that the combination of an IDH inhibitor and EGFR TKI could better inhibit lung cancer cell proliferation than an EGFR TKI alone. These findings may provide basic knowledge and valuable clues for personalized clinical management for IDH‐directed therapy in NSCLC patients.

## CONFLICT OF INTEREST

The authors declare that they have no conflicts of interest in this article. HZ, HY, XZ, YN, and TM are the employees of Hangzhou Jichenjunchuang Medical Laboratory, Co., Ltd., Hangzhou, China.

## AUTHOR CONTRIBUTIONS

SC, HZ, MJ, TM, and XL designed the research. SC, MJ, HZ, ZL, JL, and LM contributed to response evaluation and discussion of clinical significance. TL, YD, HG, CH, XZ, JL, and FL contributed to the collection of specimens. HZ led the data analysis. HZ, HY, XZ, and YN contributed to the data analysis. SC, HZ, MJ, and XZ wrote the manuscript, with input from all authors. XL supervised the whole project.

## ETHICS STATEMENT

This study was reviewed and approved by the Ethical Board at Liaoning Cancer Hospital and Institute with the ethics approval document no. 20211125. Clinical samples were used with written informed consent from all the patients.

## Supporting information


Figure S1

Figure S2

Figure S3

## Data Availability

All data needed to evaluate the conclusions in the paper are present in the paper and/or the Supplementary Materials. Original data may be made available upon request to the corresponding author.
