# Supplementary figures and images for: Molecular and clinical characteristics of IDH mutations in Chinese NSCLC patients and potential treatment strategies
Source: Cancer Med. 2022 May 8;11(22):4122–33. doi: 10.1002/cam4.4764 (PMC9678110; doi:10.1002/cam4.4764)

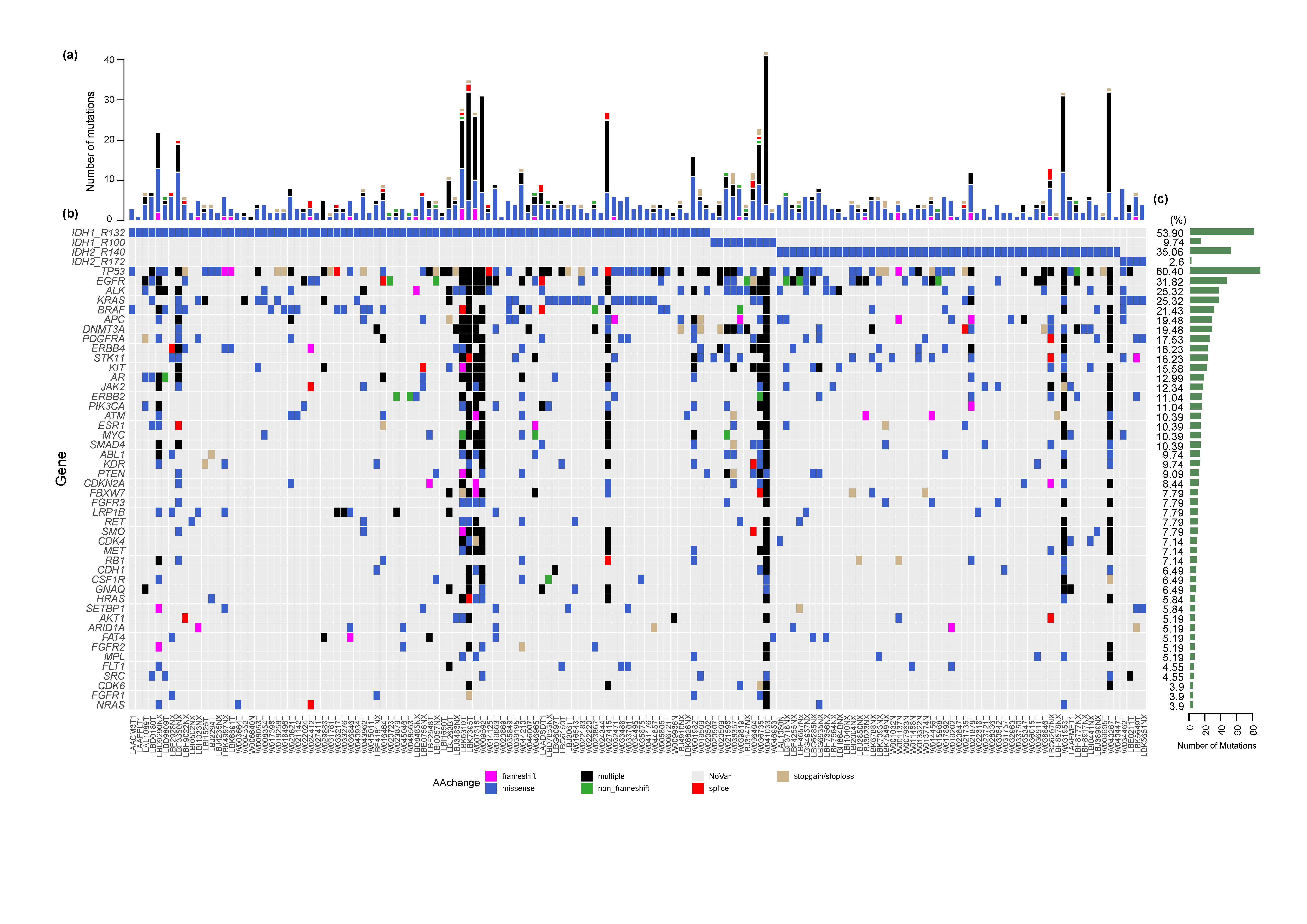

Supplement: Supplementary file 1 — Figure S1 [file CAM4-11-4122-s001.jpg]

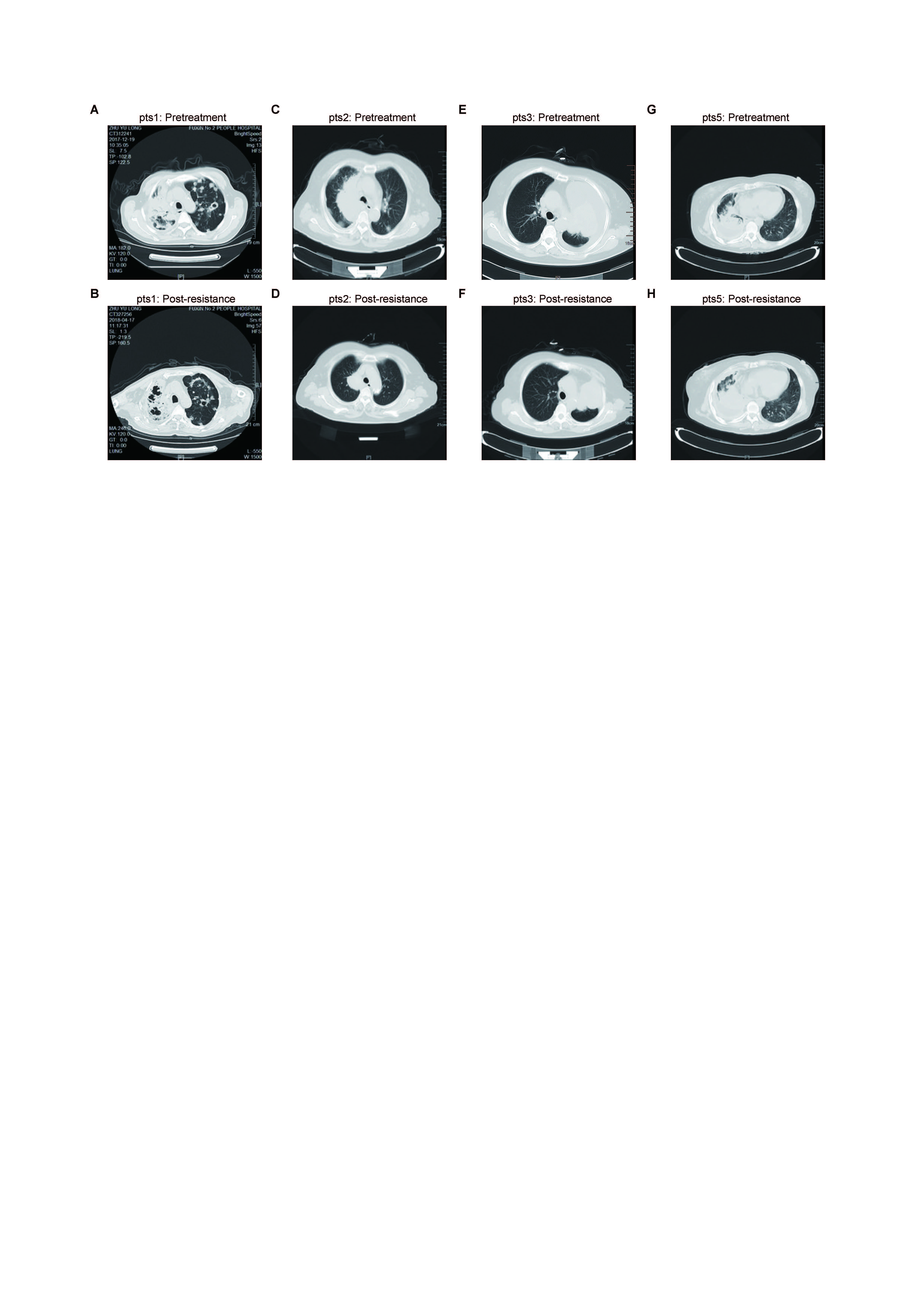

Supplement: Supplementary file 2 — Figure S2 [file CAM4-11-4122-s003.jpg]

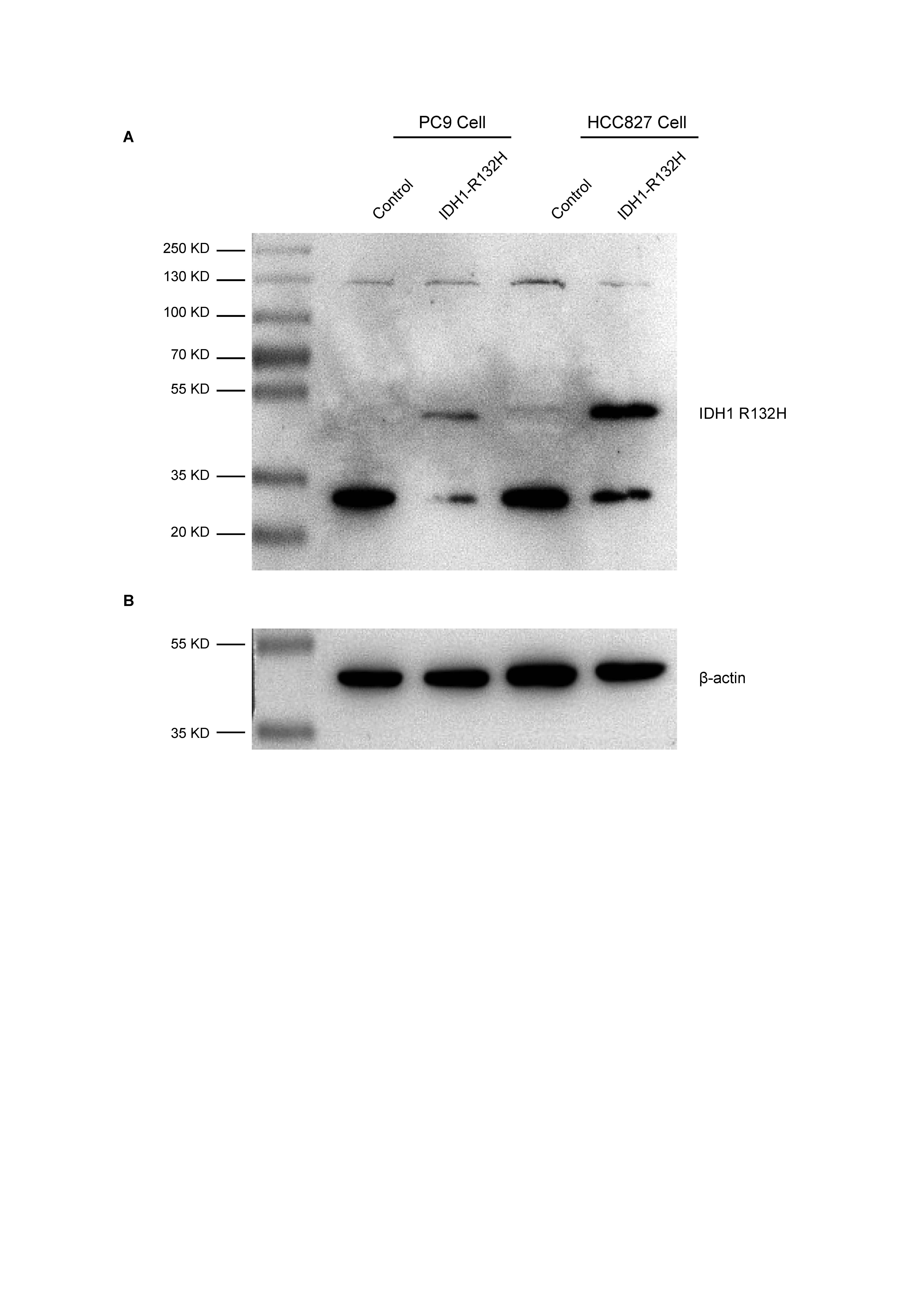

Supplement: Supplementary file 3 — Figure S3 [file CAM4-11-4122-s002.jpg]
